# Regulation of Coronaviral Poly(A) Tail Length during Infection

**DOI:** 10.1371/journal.pone.0070548

**Published:** 2013-07-29

**Authors:** Hung-Yi Wu, Ting-Yung Ke, Wei-Yu Liao, Nai-Yun Chang

**Affiliations:** Graduate Institute of Veterinary Pathobiology, College of Veterinary Medicine, National Chung-Hsing University, Taichung, Taiwan ROC; University of British Columbia, Canada

## Abstract

The positive-strand coronavirus genome of ~30 kilobase in length and subgenomic (sg) mRNAs of shorter lengths, are 5’ and 3’-co-terminal by virtue of a common 5’-capped leader and a common 3’-polyadenylated untranslated region. Here, by ligating head-to-tail viral RNAs from bovine coronavirus-infected cells and sequencing across the ligated junctions, it was learned that at the time of peak viral RNA synthesis [6 hours postinfection (hpi)] the 3’ poly(A) tail on genomic and sgmRNAs is ~65 nucleotides (nt) in length. Surprisingly, this length was found to vary throughout infection from ~45 nt immediately after virus entry (at 0 to 4 hpi) to ~65 nt later on (at 6 h to 9 hpi) and from ~65 nt (at 6 h to 9 hpi) to ~30 nt (at 120-144 hpi). With the same method, poly(U) sequences of the same lengths were simultaneously found on the ligated viral negative-strand RNAs. Functional analyses of poly(A) tail length on specific viral RNA species, furthermore, revealed that translation, *in vivo*, of RNAs with the longer poly(A) tail was enhanced over those with the shorter poly(A). Although the mechanisms by which the tail lengths vary is unknown, experimental results together suggest that the length of the poly(A) and poly(U) tails is regulated. One potential function of regulated poly(A) tail length might be that for the coronavirus genome a longer poly(A) favors translation. The regulation of coronavirus translation by poly(A) tail length resembles that during embryonal development suggesting there may be mechanistic parallels.

## Introduction

The~30 kilobase positive-strand coronavirus genome and subgenomic (sg) mRNAs of shorter lengths are 5’ capped and 3’ polyadenylated as are most eukaryotic mRNAs [1]. They also share a common leader sequence (65 to 90 nucleotides (nt) in length, depending on the coronavirus species) and a common 3’ untranslated region (~300 nt). In the group 2 mouse hepatitis coronavirus (MHV), the poly(A) tail on the genome has been determined to be about 90 nt in length using RNase T1 followed by ion-exchange chromatography and gel electrophoresis [2–5], whereas for MHV no measurement of the complementary poly(U) on the negative strand has yet been reported. In the bovine coronavirus (BCoV) genome, a 3’-terminal poly(A) tail of 60 nt and an oligo(U) tract of 8 to 20 nt have been identified by primer extension followed by head-to-tail ligation of synthesized cDNA molecules, PCR amplification and sequencing [5]. How the long poly(A) tract is generated from a short oligo(U) tract has not been described, although it has been postulated to result from a stuttering mechanism by the viral RNA-dependent RNA polymerase during synthesis of genomic or sgmRNA [5], or by a cellular cytoplasmic poly(A) polymerase [5].

Here, we report studies using a previously-described technique of head-to-tail ligation of intracellular coronaviral positive-strand RNAs and negative-strand RNAs which demonstrate that both the poly(A) on the positive-strand RNAs and poly(U) on the negative-strand RNAs can reach a length of ~65 nt, and that both, surprisingly, vary in length during infection suggesting there is a regulatory mechanism [6]. To our knowledge, the regulation of poly(A) and poly(U) lengths during infection has not been previously documented in any positive-strand poly(A)-containing RNA virus. A well-studied example of cytoplasmic regulation of poly(A) tail length occurs in oocyte mRNAs. In the cytoplasm of oocytes, certain mRNAs have relatively short poly(A) tails (20-40 nt) and are translationally inactive. During maturation, the poly(A) tail is elongated to nearly 150 nt by cytoplasmic polyadenylation and the mRNAs become translationally active [7–9]. Experimental evidence from the current study suggests that coronavirus poly(A) tail length is causally related to RNA translation efficiency. Based on these findings, we propose a process for how coronaviral poly(A) and poly(U) lengths may undergo variation and contribute to the regulation of translation and replication during infection possibly by different mechanisms.

## Results

### Poly(A) tail length on total BCoV positive-strand RNA varies during infection

**Figure 1:**
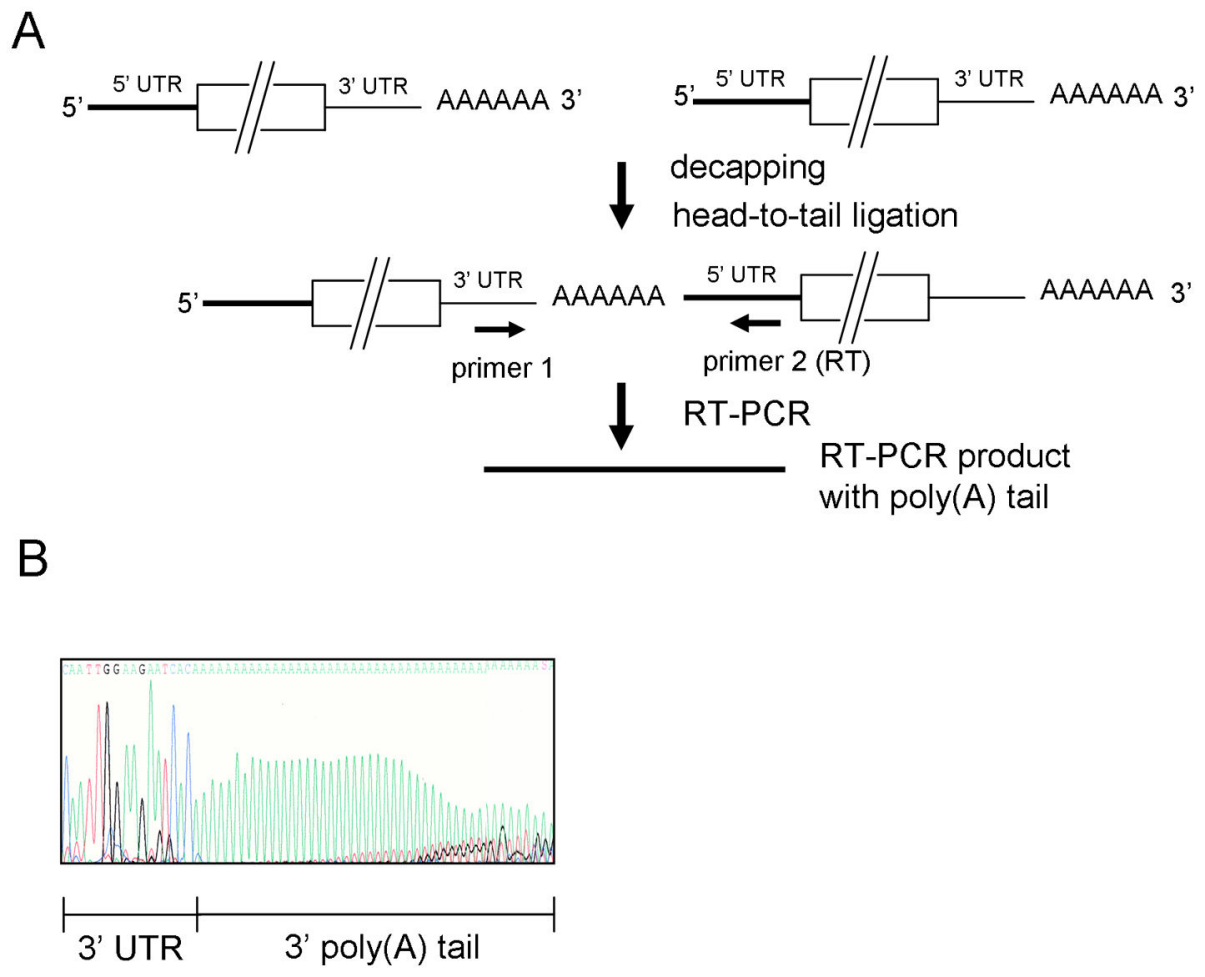
Determination of coronaviral poly(A) tail length in virions used for inoculation. (A) Strategy for determining coronaviral poly(A) tail length in virus used for inoculation. RNA extracted from BCoV harvested at 48 hpi was decapped and head-to-tail ligated. BCoV 5’ UTR-positive-strand-specific primer 2 (for RT) and BCoV 3’ UTR-negative-strand specific primer 1 were used for RT-PCR with the ligated RNA as template. Note that the head-to-tail ligation could be inter-molecular or intra-molecular. Inter-molecular-ligated RNA is represented here and in the following figures. The amplified RT-PCR product was sequenced to determine poly(A) tail length. (B) Sequence of the amplified RT-PCR product. The poly(A) tail length in virus used for inoculation was ~45 nt.

The ability to ligate positive-strand viral RNAs head-to-tail and determine poly(A) tail length soon after viral infection has enabled us to measure poly(A) tail length differences as a function of time post-infection (Figure 1A) [6,10]. To characterize these changes, we first determined poly(A) tail length by the same method in the genomes of virus used for inoculum. For this, virus inoculum was prepared in confluent human adenocarcinoma (HRT-18) cells using an MOI of ~1 virus per cell and harvesting supernatant fluids at 48 hours postinfection (hpi). RNA extracted from pelleted BCoV was decapped with tobacco acid pyrophosphatase and the 3’ end of the positive-strand viral RNA was ligated to the 5’ end of the positive-strand viral RNA with T4 RNA ligase I as described in Materials and Methods. With the use of a PCR primer set that specifically binds within 5’ and 3’ UTRs of BCoV genome (Figure 1A), RT-PCR was carried out and the resulting products were sequenced to determine the precise viral poly(A) tail lengths. The results show that the poly(A) tail length in the virus contained in the inoculum was ~45 nt (Figure 1B).

**Figure 2:**
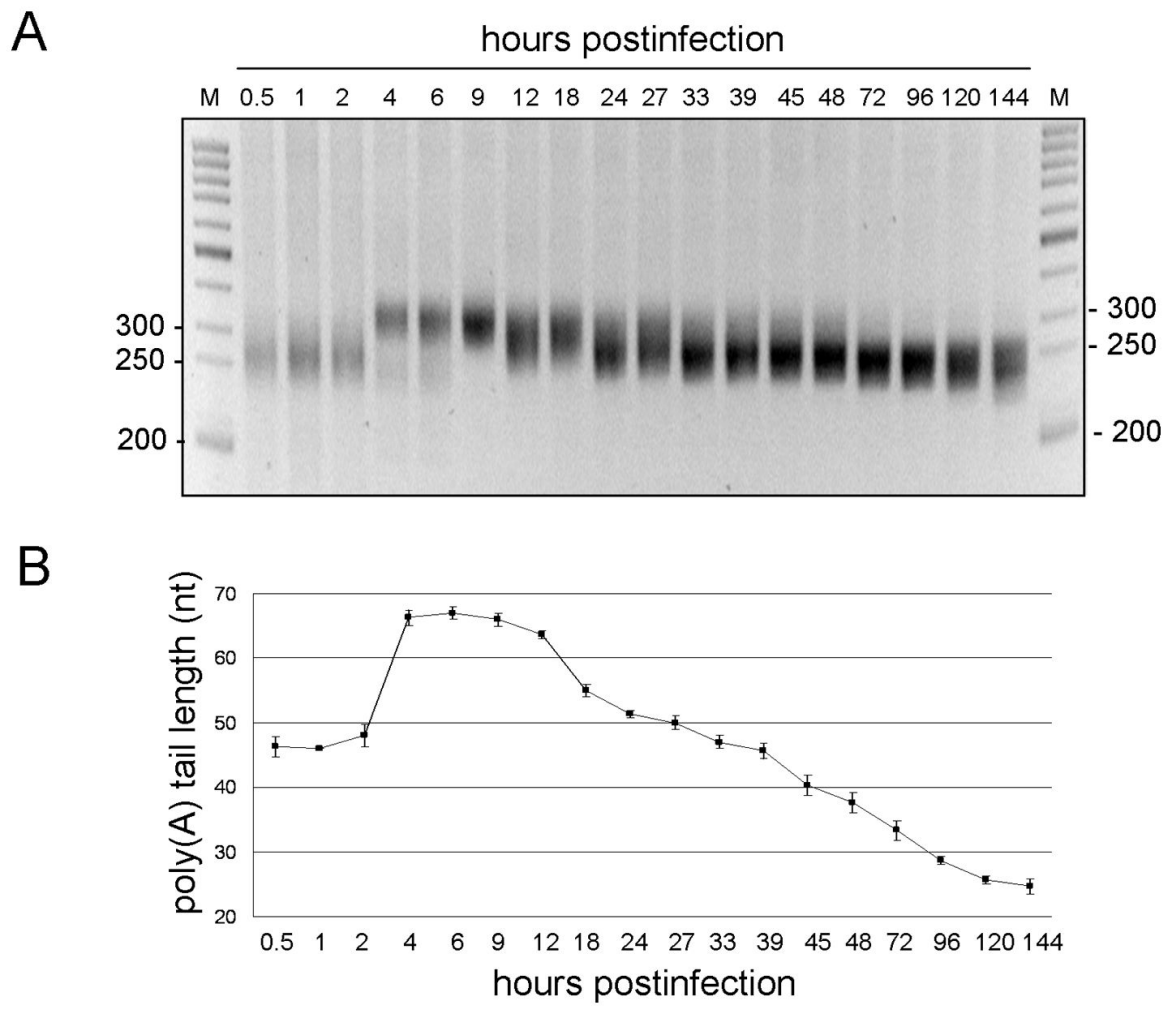
Poly(A) tail length on intra-cytoplasmic coronaviral positive-strand RNA at different times post-infection. (A) RT-PCR product synthesized from ligated RNA by the method described in Figure 1A for cytoplasmic RNA extracted from infected cells at the indicated times post-infection. RT-PCR products ranging in length from ~250 bp to ~300 bp were observed. (B) Plot of the poly(A) tail lengths as determined by sequencing RT-PCR products of ligated ends described in (A). M, ds DNA size markers in nt pairs. Values in (B) represent the mean±SD of three individual experiments.

To determine poly(A) tail length on total intracellular viral RNA as a function of time post-infection, the characterized inoculum containing an infectious titer of ~10^6^ PFU/ml was used to infect freshly-confluent HRT-18 cells with an MOI of ~10 viruses per cell and total cellular RNA was extracted at the time points indicated in Figure 2A. Total RNA was used for head-to-tail ligation and viral poly(A) tail length was determined as described above. As shown in Figure 2A, the length of RT-PCR products ranged from ~250 to ~300 base pairs (bp) and displayed an increasing then decreasing size pattern throughout the 144 h period of infection. Sequencing showed the length of viral poly(A) represented in the major population of viral RNA varied from ~45 nt early in infection (0-2 hpi) to ~65 nt later (4-12 hpi) and then gradually decreased to ~40 nt over time (12-48 hpi) (Figure 2B). The length of viral poly(A) tail became ~<30 nt after 144 h of infection. The data together suggest that the poly(A) tail length on total positive-strand BCoV RNA is regulated during infection.

### The 5’-terminal poly(U) length on total BCoV negative-strand (antigenome and sg negative strand) RNA co-varies nearly in size with the 3’ poly(A) tail on total positive-strand RNA

**Figure 3:**
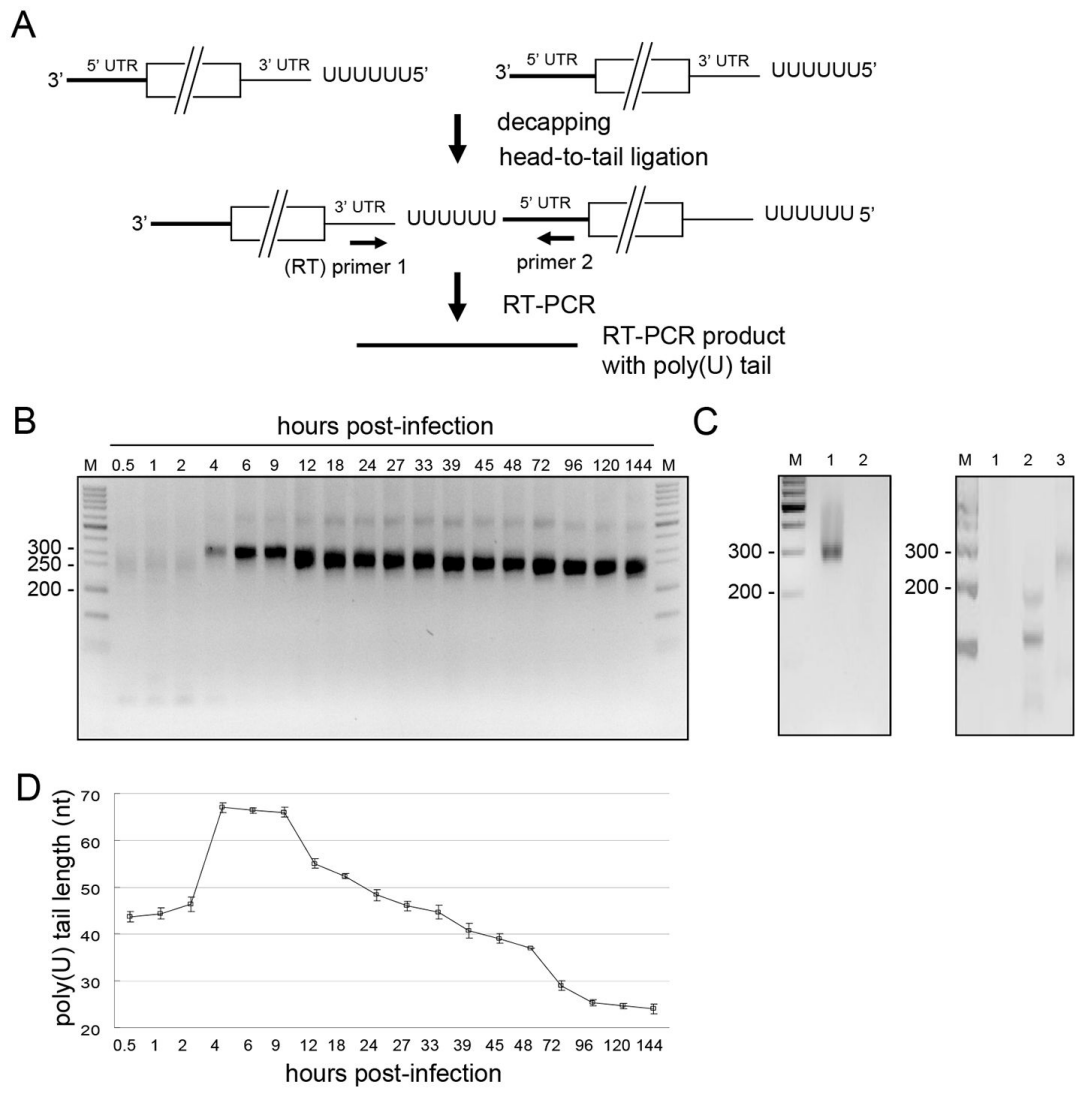
Poly(U) tract length on intra-cytoplasmic coronaviral negative-strand RNA at different times post-infection. (A) Strategy for determining coronaviral poly(U) tract length. The strategy used was the same as that described in Figure 1A except the primer used for RT was primer 1. The RT-PCR products were used to determine coronaviral poly(U) tract length. (B) Determination of RT-PCR product sizes. Varying lengths of RT-PCR products ranging from ~250 bp to ~300 bp were observed at different times post-infection. (C) RT-PCR control reactions. Left panel: RT-PCR reveals a ~300 bp product with RNA from BCoV-infected cells extracted at 9 hpi (lane 1), but not with a mixture of RNA from mock-infected cells (800 ng), from *in vitro* synthesized subgenomic mRNA 7 transcripts (100 ng), and from *in vitro* synthesized transcripts representing full-length BCoV genomic RNA (100 ng) (lane 2). Right panel: RT-PCR reaction mixture containing RNA from BCoV-infected cells at 9 hpi along with no exogenous primers (lane 1). RT-PCR reaction mixture containing RNA from BCoV-infected cells at 9 hpi along with a single primer that binds β actin mRNA (lane 2). Samples from a complete reaction with RNA from infected cells at 9 hpi served as a size reference (Figure 3C, left panel, lane 1, and Figure 3C, right panel, lane 3). (D) The length of poly(U) tracts at different times post-infection as determined by sequencing RT-PCR products obtained from samples used for panel (B). M, ds DNA size markers in nt pairs. Values in (D) represent the mean±SD of three individual experiments.

The mechanisms by which the coronaviral poly(A) tail and poly(U) tract are made are unknown. Considering that a difference in the kinetics of appearance between them might yield a clue as to how they are synthesized, the extent and timing of antigenomic poly(U) synthesis were measured and compared with the appearance of poly(A). For this, the same primers were used as for poly(A) measurement but in reverse order (Figure 3A). A major RT-PCR product of between 250 and 300 nt was found throughout infection (Figure 3B), but was not found in a control reaction containing the same primers with a mixture of *in vitro*-ligated components from mock-infected cell RNA mixed with *in vitro* synthesized positive-strand subgenomic mRNA 7 transcripts and *in vitro* synthesized transcripts representing full-length BCoV genomic RNA (Figure 3C, left panel, lane 2). Nor was a product of this size found from a control reaction mixture containing RNA from infected cells at 9 hpi but no exogenously-added primer for the RT-PCR (Figure 3C, right panel, lane 1), or a reaction mixture containing RNA from infected cells at 9 hpi but only primer that binds cellular β actin mRNA for the RT-PCR (Figure 3C, right panel, lane 2). Samples from a complete reaction with RNA from infected cells at 9 hpi served as a size reference (Figure 3C, left panel, lane 1, and Figure 3C, right panel, lane 3). In the experiment depicted in Figure 3B, a faint band migrating with an apparent size of ~400 nt was also found from 4 through 144 hpi, but when gel-purified, cloned and sequenced, it was found to contain the same sequence as the major band and thus appears to be a multimer of unknown structure. These results together suggested the ~250 to 300-nt RT-PCR product was specifically derived from negative-strand viral RNA. Interestingly, the poly(U) sequence length pattern was remarkably similar to that for poly(A) on the positive strand except that the peak length of 65 nt began to decline earlier (9 hpi for poly(U) vs 12 hpi for poly(A)) (compare Figure 2B with Figure 3D). Therefore, the poly(U) tract length was ~40 nt during the first 2 h of infection, increased to ~65 nt from 4–9 h of infection, and then decreased to ~50 nt by 24 hpi, and ~25 nt by 144 hpi. These results suggest that the length of coronaviral poly(U), like that of poly(A), is regulated during BCoV infection.

### The poly(A) tail length on subgenomic mRNA 7 and the poly(U) tract length on subgenomic mRNA 7 negative strand co-vary in kinetics and size with those on total viral positive and negative strands

**Figure 4:**
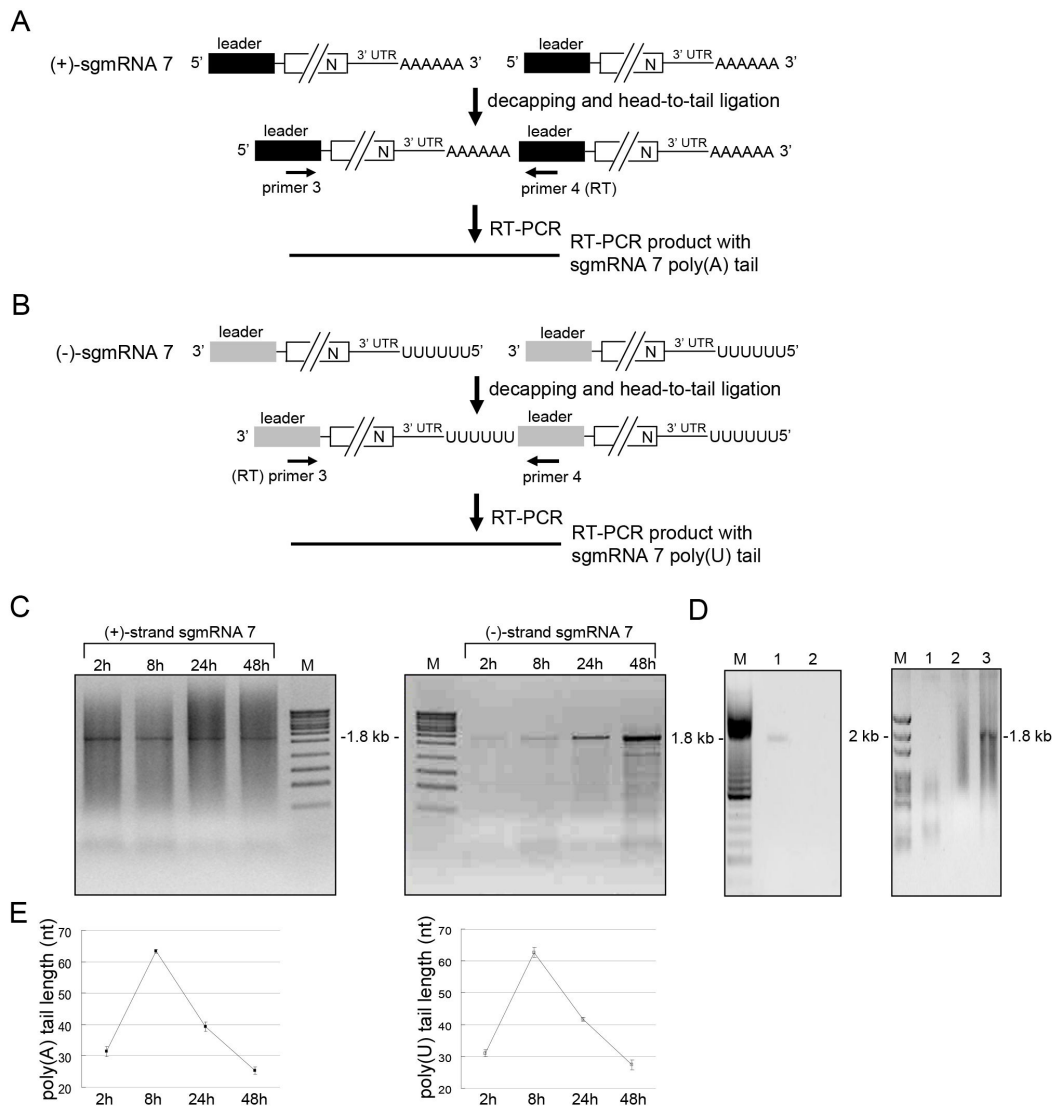
The length of poly(A) and poly(U) on subgenomic mRNA 7 positive strand and negative strand, respectively. (A) Strategy for determining poly(A) tail length on coronaviral subgenomic mRNA 7 positive strand. Total cellular RNA extracted from BCoV-infected HRT cells was decapped and head-to-tail ligated. To ensure the poly(A) tail sequence is specifically derived from subgenomic mRNA 7 rather than genomic RNA, BCoV leader positive-strand-specific primer 4 (for RT) and BCoV leader (-)-strand-specific primer 3 were used for RT-PCR with ligated positive-strand coronaviral RNA as a template. The expected length of RT-PCR product is near 1.8 kb containing subgenomic mRNA 7-specific poly(A) tail. The RT-PCR product was sequenced to determine subgenomic mRNA 7 poly(A) tail length. (B) Strategy for determining poly(U) tract length on coronaviral subgenomic mRNA 7 negative strand. The method used to determine the length of poly(U) tail on negative-strand subgenomic RNA is the same as that for poly(A) tail length on positive-strand subgenomic mRNA 7 except the primer used for RT was primer 3. The expected length of RT-PCR product which contains subgenomic mRNA 7-specific poly(U) tract is ~1.8 Kb. The RT-PCR product was sequenced to determine subgenomic mRNA 7 poly(U) tract length. (C) RT-PCR product synthesized with the methods described in panels (A) and (B). The length of subgenomic mRNA 7 RT-PCR products containing poly(A) (left panel) or poly(U) (right panel) was near 1.8 kb at the different times postinfection. (D) RT-PCR control reactions. Same as described for Figure 3C, except for the use of primers 3 and 4 (not 1 and 2) in lane 1, left panel, and lane 3, right panel. (E) The length of coronaviral subgenomic RNA 7 poly(A) tail (left panel) and subgenomic RNA 7 poly(U) tract (right panel) at different time points postinfection as determined by sequencing RT-PCR products obtained in Figure 4C. M: ds DNA size markers in nt pairs. Values (E) represents the mean±SD of three individual experiments.

To determine the poly(A) and poly(U) sequence lengths on subgenomic mRNA, advantage was taken of the greater abundance and shorter length of subgenomic mRNA 7 from its leader to the poly(A) tail (a distance of 1.8 kb vs 2.4 kb for the next-larger subgenomic mRNA, subgenomic mRNA 6, because of the coronavirus 3’ nested set arrangement of subgenomic mRNAs) [6]. RT-PCR products made in this way, specific for subgenomic mRNA 7 positive and negative strands, were separately made and sequenced [6]. This strategy to characterize the lengths of the poly(A) and poly(U) is depicted in Figure 4A and Figure 4B, respectively. RT-PCR products of ~1.8 kb for subgenomic mRNA 7 positive strand were detected (Figure 4C, left panel). RT-PCR products of ~1.8 kb for subgenomic mRNA 7 negative strand were also detected (Figure 4C, right panel). In the experiment depicted in Figure 4C, the smear of faint bands above and below the 1.8 kb fragment were of unidentifiable mixed sequence as determined by cloning and sequencing. In addition, RT-PCR products of ~1.8 kb for subgenomic mRNA 7 negative strand were found from BCoV-infected cell RNA at 8 hpi (Figure 4D, left panel, lane 1, and Figure 4D, right panel, lane 3), but not from a control reaction which used an *in vitro*-ligated mixture of mock-infected cell RNA mixed with positive-strand subgenomic mRNA 7 transcript and full-length transcripts representing full-length BCoV genomic RNA (Figure 4D, left panel, lane 2). Nor were they found from RT-PCR reaction mixtures that contained RNA from infected cells at 8 hpi but no exogenously-added primer (Figure 4D, right panel, lane 1), or that contained cellular β actin mRNA-specific primer (Figure 4D, right panel, lane 2). These results indicate that the RT-PCR product shown in Figure 4C, right panel, arose specifically from negative-strand subgenomic mRNA 7. From direct sequencing of the RT-PCR products, the lengths of poly(A) on positive-strand subgenomic mRNA 7 were determined to be ~30, ~64, ~38 and ~34 nt at 2, 8, 24 and 48 hpi, respectively (Figure 4E, left panel), and of poly(U) on subgenomic mRNA 7 negative strands to be ~32, ~61, ~41 and ~29 nt at 2, 8, 24 and 48 hpi, respectively (Figure 4E, right panel). These results show that the length of the poly(A) tail on subgenomic mRNA 7 and of the poly(U) tract on subgenomic mRNA 7 negative strands co-vary closely in kinetics and size with those on total viral positive and negative strands.

### The poly(A) and poly(U) lengths on replicating defective interfering RNA and its antigenome co-vary in length during infection

**Figure 5:**
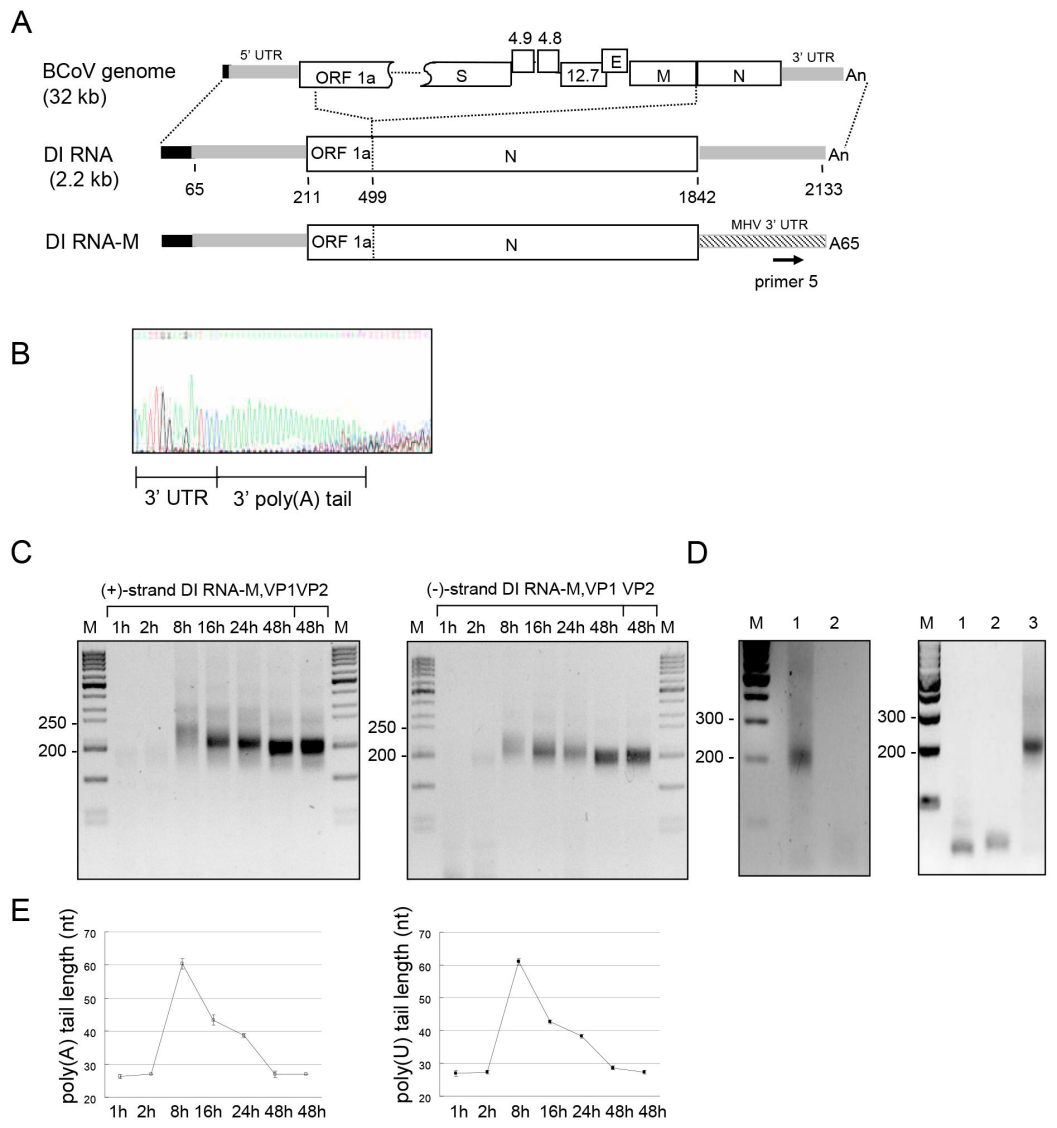
The poly(A) and poly(U) lengths on BCoV DI RNA positive strand and negative strand, respectively. (A) Diagram of BCoV DI RNA-M used to determine poly(A) and poly(U) lengths on BCoV DI RNA during coronavirus infection. To distinguish the DI RNA-specific poly(A) tail and poly(U) tract from those on BCoV helper virus RNAs, the BCoV DI RNA 3’ UTR was replaced with the MHV 3’ UTR to synthesize a previously documented replication-competent, packagable BCoV DI RNA-M [6]. The methods used to determine the DI RNA-M poly(A) and poly(U) lengths are similar to those described in Figures 2 and 3 except that primer 5 is used. This method enables discrimination between helper virus RNA and DI RNA-M as the origin of the poly(A) and poly(U). (B) Sequence of the poly(A) tail on BCoV DI RNA-M in the inoculum. The experiment described in panel (A) shows that the poly(A) tail length of BCoV DI RNA-M in the virus used for inoculation was ~26 nt. (C) RT-PCR product synthesized with the method described in panel (A). Varying lengths of RT-PCR products which contained DI RNA-M poly(A) tail (left panel) and poly(U) tract (right panel) ranged from ~200 bp to ~250 bp at different times following infection with virus passage 1 (VP1) or 2 (VP2). (D) RT-PCR control reactions. Left panel: RT-PCR with primers 5 and 2 reveals a ~200-bp product with RNA extracted from total cell RNA collected at 24 hpi with DI RNA-M-containing BCoV (lane 1), but not from a mixture of RNA extracted from BCoV-infected cells at 24 h of VP1 (100 ng) and from positive-strand DI RNA-M (100 nt) (lane 2). Right panel: A ~200-bp product was not obtained from RT-PCR reactions with RNA from cells infected with DI RNA-M-containing BCoV and no exogenous primers (lane 1), or from the same RNA with a primer that binds β actin mRNA (lane 2). (E) The length of DI RNA-M poly(A) tail (left panel) and poly(U) tract (right panel) at different time points of VP1 and at 48 h of VP2 as determined by sequencing RT-PCR products obtained in panel (C). M, ds DNA size markers in nt pairs. Values (E) represents the mean±SD of three individual experiments.

Since it is not technically feasible to apply the same strategy as was used for subgenomic mRNA 7 analysis to determine the lengths of the poly(A) tail and poly(U) tract on the viral genome and antigenome (an analysis of an RT-PCR product of >7 kb would be required), an alternative approach was used. For this, the BCoV defective interfering (DI) RNA, a helper-virus-dependent replicon of 2.2 kilobases in length (Figure 5A) [11] was used. This BCoV DI RNA is a naturally-occurring DI RNA that has been modified with a reporter and extensively analyzed with regard to required *cis*-acting elements for replication [6,12–16]. To analyze the poly(A) and poly(U) tail lengths on this molecule, the BCoV DI RNA was modified to carry the MHV 3’ UTR (which was named DI RNA-M, Figure 5A) with which a MHV-specific oligonucleotide primer for RT-PCR analysis could be used [6]. For analysis, BCoV was used as helper virus and DI RNA-M was transfected into BCoV-infected HRT-18 cells. To mimic the kinetics of BCoV natural infection, supernatant fluids containing packaged DI RNA progeny were collected from BCoV-infected DI RNA-M-transfected HRT-18 cells at 48 h posttransfection (hpt) and used as inoculum to infect fresh HRT-18 cells (designated virus passage 1, VP1) [17]. The poly(A) tail length of packaged DI RNA–M in the VP1 virus used for inoculum was determined to be ~26 nt (Figure 5B) by the same strategy as depicted in Figure 1A except that primer 1 was replaced with primer 5 which specifically binds MHV 3’ UTR (Figure 5A). After inoculation, total cellular RNA was extracted at the indicated time points for VP1 and VP2 as shown in Figure 5C. The length of the poly(A) was determined by sequencing the RT-PCR products from the ligated positive strand RNAs (Figure 5C and E, left panel). To determine poly(U) length, the same primers were used as for poly(A) measurement but in reverse order. RT-PCR products were detected (Figure 5C, right panel) and sequenced to determine poly(U) length (Figure 5E, right panel). Three control reactions were used to determine the specificity of the negative-strand RT-PCR product. Whereas a ~200-nt negative-strand RT-PCR product was found from RNA extracted from BCoV-infected cells at 24 h of VP1 (Figure 5D, left panel, lane 1 and Figure 5D right panel, lane 3), a product of this size was not found from RT-PCR reactions with an *in vitro*-ligated mixture of BCoV-infected cell RNA with positive-strand input DI RNA-M (Figure 5D, left panel, lane 2) [6]. Nor was it found in an RT-PCR reaction mixture containing RNA from cells infected with DI RNA-M-containing BCoV and no exogenous primer (Figure 5D, right panel, lane 1), or in an identical RT-PCR reaction mixture but with primer that binds cellular β actin mRNA (Figure 5D, right panel, lane 2), indicating that the measured poly(U) length (Figure 5E, right panel) was specifically from negative-strand DI RNA. The overall results, therefore, reveal that the length of both poly(A) and poly(U) was ～27 nt at early times after infection (0-2 hpi), which increased to ～62 nt at 8 hpi, and then decreased to ～28 nt at 48 hpi. Although using a BCoV DI RNA with MHV 3’ UTR could possibly alter the poly(A) and poly(U) lengths when compared with wt BCoV DI RNA, these results still indicate that the length of the poly(A) tail on the hybrid DI RNA positive strand and of the poly(U) tract in the complementary strand, which are presumably the same as on the replicating viral genome, are regulated throughout replication. Furthermore, the kinetics of the poly(A) and poly(U) changes parallel those as measured for the total RNA and sgmRNA 7 described above.

### The coronaviral poly(A) tail length on BCoV DI RNA, a surrogate for the viral genome, correlates positively with translation efficiency in BCoV-infected HRT-18 cells

**Figure 6:**
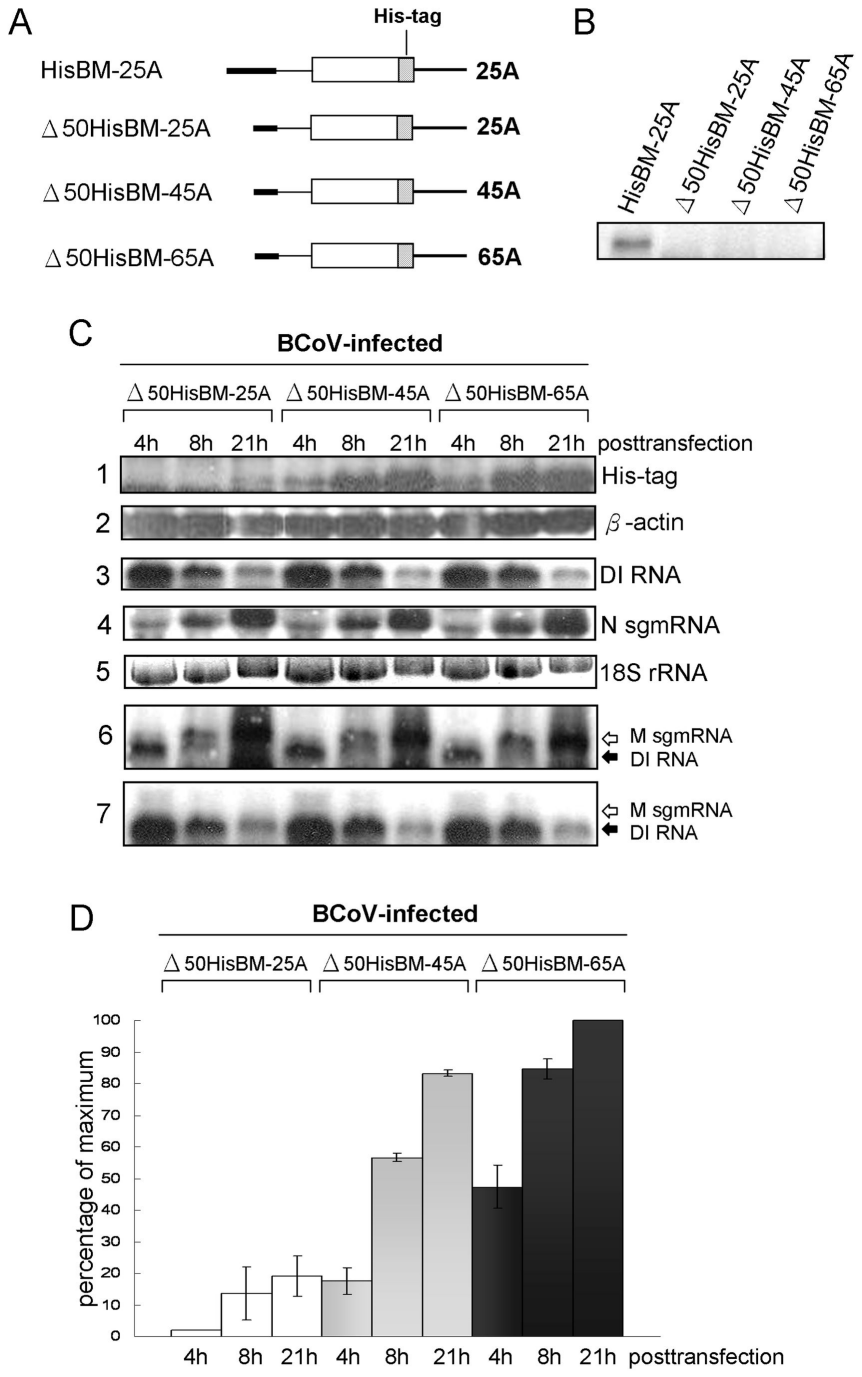
Effect of coronaviral poly(A) tail length on translation of non-replicating coronaviral DI RNA in virus infected cells. (A) DI RNA constructs used for replication and translation assay. The open box represents a single, 2.2 kb open reading which is followed by a stippled box representing an in-frame 18-nt His-tag coding region. (B) Replication of DI RNAs. A Northern blot of RNAs made at 48 hpi following infection with VP1 to determine the replication level of mutated DI RNAs. Lanes 1 through 4 show the accumulation of DI RNA. (C) Abundance of non-replicated DI RNA and DI RNA-expressed proteins. Expression of His-tagged DI RNA protein was measured by Western blot analysis with antibody specific to the Histidine-tag. BCoV-infected HRT cells were transfected with the named DI RNA at 2 hpi and at the indicated times proteins or RNAs were extracted for analysis. Protein samples from cell lysates were harvested at 4, 8, and 21 hpt and the His-tagged protein (row 1) and cellular β actin (row 2) were measured by Western blotting. For RNA measurements, RNA was extracted at the indicated times and Northern probing was done with the TGEV reporter probe to measure DI RNA levels (row 3), with intra-N subgenomic mRNA probe to measure subgenomic N mRNA levels (row 4), subgenomic M mRNA and DI RNA levels (row 6), with 18S rRNA-specific probe to measure 18 S rRNA levels (row 5), and with an oligo-histidine-specific probe to detect His-tag coding sequence in viral subgenomic RNA and His-tagged DI RNA (row 7). (D) Quantification of translated protein from individual DI RNA constructs at different time points. Values (D) represent the mean±SD of three individual experiments.

The link between coronaviral poly(A) tail and translation has not been previously described. On eukaryotic mRNAs, a widely-documented behavior of the 3’-terminal poly(A) tail is that an increase in its length positively correlates with an increased translation efficiency [18,19]. Such polyadenylation in oocytes, for example, activates translation of dormant mRNAs with short poly(A) tails. Therefore, one possible function of the increasing poly(A) tail length on the coronavirus genome during replication is a condition-dependent enhancement of translation. To test the hypothesis that increasing poly(A) tail length on the viral genome results in increased translation efficiency, BCoV DI RNA constructs with a replication-blocking 5’-terminal 50-nt deletion [17] but with differing poly(A) tail lengths (Figs. 6A and 6B) were tested. The rationale for this experiment was twofold: (i) Poly(A) tails with distinct lengths of 25, 45, and 65 nt were found on replicating DI RNAs in infected cells (Figure 5E, left panel) (ii). Using replication-incompetent DI RNAs enables an assessment of translation only from transfected DI RNAs with defined poly(A) tail lengths. To determine whether poly(A) tails of differing lengths affect translation efficiency of DI RNA in infected cells, replication incompetent His-tagged BCoV DI RNAs with lengths of 25, 45 and 65 nt were produced *in vitro* and studied *in vivo*. For this, equal amounts (3µg) of transcript were transfected into BCoV-infected HRT cells and accumulation of the DI RNA-encoded protein was measured by Western blot analysis (Figure 6C, row 1) and compared with the steady-state expression of cellular β actin (Figure 6C, row 2). The amount of His-tagged protein increased markedly over time with poly(A) tails of 45 and 65 nt, and less so with a poly(A) tail of 25 nt (Figure 6C, row 1, and summarized in Figure 6D). To relate these quantities to the abundance of DI RNA template and to three other RNA species, Northern analyses were carried out on electrophoretically-separated RNA in samples from the same experiment (Figure 6C, rows 3 through 6). The His-tag-encoding replication-defective BCoV DI RNA was measured with a probe specific for the 30-nt reporter sequence in BCoV DI RNA (TGEV(+)) (Figure 6C, row 3) and was found to decrease in abundance for DI RNAs with all three poly(A) tail lengths (Figure 6C, row 3), whereas the N-encoding sgmRNA 7 from helper virus, as measured by an N-specific probe, steadily increased over time in the presence of all three DI RNA constructs (Figure 6C, row 4), and 18S rRNA remained relatively steady throughout (Figure 6C, row 5).

One interpretation of these results is that the protein is synthesized more efficiently from constructs with longer poly(A) tails, and although the RNA template from the non-replicating DI RNA degraded with time, the accumulating His-tagged protein persisted in the cell because of a longer half-life. Another interpretation is that the His-tag coding sequence could have recombined with the helper virus genome and become expressed from helper virus sgmRNAs containing this sequence. Recombination of this nature has been demonstrated when experimentally-applied selective pressures are used [20]. To seek evidence of recombination, Northern analysis after infection and transfection was carried out to detect the His-tag-coding sequence in the helper virus genome and subgenomic mRNAs. No evidence of His-tag coding sequence was found in any of the viral sgmRNAs or genome within the 21 h infection period that could explain the expression of the His-tagged protein from this source (Figure 6C, rows 6 and 7, and data not shown). An increase in accumulated His-tagged protein in the presence of a decreasing DI RNA abundance is, therefore, apparently due to a longer half-life of the protein molecule. Therefore, the enhancement of viral translation efficiency by a longer coronavirus poly(A) tail suggests that poly(A) tail length plays a role in the regulation of coronaviral genome translation.

## Discussion

In this study we report that the coronaviral 3’-terminal poly(A) tail length in total viral RNA, sgmRNA7, and DI RNA is relatively short (~26-45 nt) in infected cells at 0-2 hpi, increases to peak length (~65 nt) at ~6-10 hpi, and gradually decreases in size (~30-45 nt) after ~10 h of infection. An analysis of poly(A) sequences in 16 isolated bacterial colonies at 39 hpi showed tail lengths to range from 39 to 45 nt, suggesting the mean sizes measured by group sequencing is a good reflection of lengths in the major population at the times measured. This finding appears to represent a regulation of polyadenylation in a coronavirus that mimics aspects of cytoplasmic regulation of poly(A) tail length in certain mRNAs during the maturation of oocytes. It suggests also that there may be a regulated control of poly(A)-influenced translation. This behavior, to our knowledge, has not been previously documented in coronaviruses nor in other poly(A) tail-containing positive-strand RNA viruses, although poly(A) addition following transfection of non-polyadenylated MHV MIDI-C defective interfering (DI) RNAs into infected cells has been reported [21]. We also report that the length of 5’-terminal poly(U) on coronaviral negative-strand RNA co-varies similarly in length in a regulated fashion with the poly(A) tail. These findings have led us to reexamine previously proposed mechanisms for coronaviral poly(A) and poly(U) synthesis [5] and to speculate on the role of these differences in coronavirus replication.

The coronaviral poly(U) length at the 5’ end of negative-strand RNA in BCoV as reported here differs from two previous reports [5,6]. (i) In the first [5], the poly(U) on BCoV negative-strand RNA was measured at 8-20 nt in length at 24 hpi vs ~45 nt at 24 hpi as described here. We suggest that the shorter sequence resulted from an experimental approach that favored selection of shorter sequences. In the method used, a radiolabeled oligodeoxynucleotide primer was annealed near the common 5' end of the negative-strand RNAs, extended with reverse transcriptase, ligated head-to-tail, amplified by the PCR, cloned, and sequenced. This method would have favored enrichment of the shorter extended products owing to the secondary structures in the RNA template that contribute to reverse transcriptase pausing, and perhaps also RNA degradation [22–24]. (ii) In the second it was reported that the poly(U) tract was 30 nt at 12 hpt on the negative-strand of replicating DI RNA in virus-infected cells [6]. In the current study, we used this latter method but with modifications. Most notably, the poly(U) of >40 nt was observed prior to 12 hpi (Figure 3A) and was measured on a replicating DI RNA that had gained entry into cells as a packaged DI RNA in the infecting virion, not as a transfected RNA. We speculate that gaining entry via infection may have presented the DI RNA a more favorable microenvironment for replication.

It has been suggested that influenza virus employs a stuttering mechanism in which viral RNA polymerase pauses at a stretch of short U residues located at the 5’ terminus of the (-)-strand viral genomic RNA and moves back and forth over this stretch of U residues to synthesize the poly(A) tail [25–27]. It has been proposed that the coronavirus also may exploit a stuttering mechanism to generate a viral poly(A) tail from a short poly(U) tract [5]. It might also be possible that the coronaviruses uses an AAUAAA signal or its variations in the 3’ UTR for cytoplasmic polyadenylation [28,29], or alternatively a Musashi protein-dependent polyadenylation as the result of a element of (G/A) U_1−3_AGU [30]. However, the current findings that (i) the length of coronaviral poly(U) tract is almost the same as the poly(A) tail (~65 nt), and that (ii) the poly(U) and poly(A) tracts display the same pattern of increased-then-decreased length during infection (Figure 2B, and Figure 3D) lead us to propose an alternate mechanism for polyadenylation and uridylation which is as follows: (i) As with poliovirus [31], coronaviruses use full-length homopolymeric stretches in the positive- and negative-strands for the addition of poly(A) tail and poly(U) tracts at the same times during infection (ii). Lengthening of the poly(A) and poly(U) stretches prior to 12 hpi may be carried out by a stuttering mechanism, although at this time we have no evidence of such a mechanism (iii). The shortening of the poly(A) or poly(U) tracts after 12 hpi may be a function of BCoV replicase or a cellular deadenylase [32]. Taken together, the evidence indicates that coronaviruses may employ more than one mechanism for the regulation of poly(A) and poly(U) lengths during infection.

It has been demonstrated that the regulation of polyadenylation in the cytoplasm for specific mRNAs during oocyte maturation is associated with the regulation of translation [7–9]. In this study, our experimental evidence also shows that coronaviral poly(A) tail length is positively correlated with translation efficiency suggesting translation may be likewise regulated by coronaviral poly(A) tail length. However, unlike cellular mRNA which is only used as a template for translation, the coronavirus positive-strand RNA genome must serve as a template not only for translation but also for negative-strand RNA synthesis. Since (i) translation and negative-strand RNA synthesis cannot occur simultaneously on the same template and (ii) the longer coronaviral poly(A) tail length is preferred for translation, we speculate that one benefit of the regulated poly(A) tail length during coronavirus infection might be that it functions to regulate the processes of viral genome translation and negative-strand RNA synthesis. On the other hand, since BCoV DI RNA with the longer poly(A) tail replicated better [21] and the length of the poly(U) tract is essentially the same as that of the poly(A) tail at the same time (this study), we suggest that the regulated poly(U) length on negative-strand RNA is a critical factor regulating replication. Accordingly, regulation of poly(A) and poly(U) length may function in regulating viral translation and replication as required by the virus for replication. Further studies are needed to test these possibilities.

## Materials and Methods

### Plasmid constructs

Construction of pDrep1 and pDI RNA-M (formerly called pDI RNA-2) [6] which encodes BCoV DI RNA and DI RNA-M, respectively, and were kindly provided by David Brian (University of Tennessee, Knoxville, TN), has been described [6,17]. In brief, pDI RNA-M was made by replacing the 288-nt 3’ UTR of BCoV-Mebus in pDrep1 with the 301-nt 3’ UTR of MHV-A59 (GenBank accession no. NC_001846) from fragment G DNA [33].

To construct pHisBM-25A, pΔ50HisBM-25A, pΔ50HisBM-45A and pΔ50HisBM-65A, an overlap PCR mutagenesis procedure was used as described [34], but with the appropriate sets of oligonucleotides for poly(A) tail lengths of 25, 45, or 65 As. The overlapping PCR product was cloned into TOPO-XL vector (Invitrogen) and digested with *Xba*I and *Mlu*I. The digested fragment was cloned into *Xba*I- and *Mlu*I-linearized pDrepI to make pHisBM-25A or pD50 to make pΔ50HisBM-25A, pΔ50HisBM-45A and pΔ50HisBM-65A [17].

Six cDNA clones representing the full length of the BCoV-Mebus genome, and pNrep1 which encodes the entire subgenomic mRNA 7 [17] were kindly provided by David Brian (University of Tennessee, Knoxville, TN).

### Preparation of viral RNA and DI RNA from infected cells

The Mebus strain of BCoV (GenBank accession no. U00235) was plaque-purified three times and grown on a HRT-18 cell line as described [4,35]. BCoV at 3 × 10^7^ PFU/ml to be used for inoculum was prepared by passaging virus three times at a multiplicity of infection 1 PFU per cell. To prepare RNA for determining coronaviral poly(A) and poly(U) lengths, HRT-18 cells at approximately 5 × 10^6^ per 35-mm-diameter dish when 100% confluent were infected with BCoV at a multiplicity of 10 PFU per cell. Total cellular RNA was extracted with TRIzol (Invitrogen) at the different time points post-infection as indicated in each experiment. To prepare coronaviral RNA from purified virus, supernatant from BCoV-infected HRT-18 cells at 48 hpi. was collected and extracted with TRIzol (Invitrogen).

To identify the poly(A) tail length on DI RNA-M in packaged virus, supernatant fluids from BCoV-infected and DI RNA-M transfected HRT-18 cells at 48 hpt were collected and extracted with TRIzol (Invitrogen). To prepare RNA for identifying poly(A) and poly(U) lengths on DI RNA-M in infected cells, HRT-18 cells at approximately 4 × 10^6^ per 35-mm-diameter dish at 80% confluency were infected with BCoV (as helper virus) at a multiplicity of 10 PFU per cell and 500 ng of DI RNA-M transcript, obtained as T7 RNA polymerase transcripts of pDI RNA-M linearized at the *Mlu*I site [immediately downstream of the poly(A) tail], was transfected into BCoV-infected cells with the use of Lipofectin (Invitrogen) as previously described [36]. Supernatant fluids were harvested at 48 hpt and 500 µl was used to infect freshly confluent HRT-18 cells in a 35-mm dish (virus passage 1, VP1). Total cellular RNA was extracted with TRIzol (Invitrogen) at the indicated time points.

### Head-to-tail ligation of viral RNA and RT-PCR reactions to determine poly(A) and poly(U) tail lengths

A head-to-tail ligation method previously used to identify terminal features on influenza virus [10] and coronavirus [6] RNAs was employed here with some modifications to determine poly(A) and poly(U) tail lengths. In brief, to determine the poly(A) tail length, 10 µg of extracted total cellular RNA in 25 µl of water, 3 µl of 10X buffer and 10 U of (in 1 µl) tobacco acid pyrophosphatase (Epicentre) were used to de-block the 5′ capped end of the genomic RNA. Following decapping, phenol-chloroform-extracted RNA in 25 ul of water was heat-denatured at 95°C for 5 min and quick-cooled. Then 3 µl of 10X ligase buffer and 2U (in 2 µl) of T4 RNA ligase I (New England Biolabs) were added, and the mix was incubated for 16h at 16°C. For the RT reaction, SuperScript II reverse transcriptase (Invitrogen), which transcribes poly(A) tails and poly(U) tracts of greater than 100 nt with fidelity was used as previously described [37,38]. For RT-PCR, phenol-chloroform-extracted ligated RNA was used for the RT reaction and the resulting cDNA was used for PCR with AccuPrime Taq DNA polymerase (Invitrogen). To determine the length of poly(A) tail on positive-strand coronaviral RNA, primer 2, which binds nt 107–129 from the 5’ end of BCoV positive strand, was used for RT, and for the PCR, 5 μl of the resulting cDNA mixture was used in a 50-µl PCR with primers 2 and 1, which binds nt 87–110 from the poly(U) tail on the negative strand of the BCoV 3’ UTR. To determine the length of the poly(U) tails on coronaviral RNA, the same primers were used but in reverse order. To determine the poly(A) and poly(U) tail lengths on subgenomic mRNA 7 from BCoV-infected cells, BCoV leader positive-strand-specific primer 4, which binds nt 8-27 of leader sequence from 5’ end of BCoV positive-strand subgenomic mRNA 7, and BCoV leader negative-strand-specific primer 3, which binds nt 29–54 of leader sequence from 3’ end of BCoV negative-strand subgenomic mRNA 7, respectively, were used for RT. Five µl of resulting cDNA was used in a 50-µl PCR with primer 3 and primer 4 (Figure 4A and 4B). To examine the DI RNA-M poly(A) and poly(U) tail lengths, primer 6, which binds nt 29–54 of leader sequence from 5’ UTR of BCoV positive strand, and primer 5, which binds nt 99–122 from the poly(U) tail on the negative strand of the MHVA59, respectively, were used for RT. Five µl of resulting cDNA was used in a 50-µl PCR with primers 5 and 6. Non-viral RNA target primer used for control reactions was one that binds β actin mRNA (β actin(+): 5’ CAAAGGCGAGGCTCTGTGCTCGC3’). The resulting 50-µl PCR mixture was heated to 94°C for 2 min, then subjected to 34 cycles of 30 s at 94°C, 30 s at 55°C, and 30 s at 72°C. The predicted poly(A) or poly(U)-containing PCR product was sequenced to determine the length of poly(A) or poly(U) tail.

### Western blot analysis for *in vivo* translation of DI RNA

*Mlu*I-linearized plasmid DNA constructs Δ50HisBM-25A, Δ50HisBM-45A and Δ50HisBM-65A were transcribed *in vitro* with the mMessage mMachine T7 transcription kit (Ambion) in a total reaction volume of 50 µl supplemented with 7.5 µl of 30 mM GTP. *In vitro* transcription was done at 37°C for 60 min, treated with 5 µl Turbo DNAse (Ambion) at 37°C for 30 min, and chromatographed through a Biospin 6 column (Bio-Rad) before use in transfection [16]. For transfection, HRT-18 cells in 35-mm dishes at ~80% confluency (~8 × 10^5^ cells/dish) were first infected with BCoV at a multiplicity of infection of 5 PFU per cell and then transfected at 2 hpi with 3 µg of transcript RNA using Lipofectin (Invitrogen) [17]. Protein samples from cell lysates were harvested from HRT-18 cells at the time points indicated in Figure 6B, electrophoresed on 12% SDS-PAGE gels, and electrotransferred to a nitrocellulose membrane (Amersham Biosciences). Proteins of interest were detected by Western analysis using primary antibody specific to Histidine tag (Serotec) or β actin (Serotec) as primary antibody, and Goat anti mouse IgG conjugated HRPO as secondary antibody (Jackson). Proteins were visualized by Western Lightning™ Chemiluminescence Reagent (PerKinElmer NEL105) and X-ray film (Kodak) [39].

### Northern assay for intracellular DI RNA, helper virus RNA, N subgenomic mRNA, His-tag-encoding sequence, and 18S rRNA

The Northern assay for detecting reporter-containing DI RNAs was performed as described previously [16,17]. Briefly, 3 µg of DI RNA transcript was transfected into HRT cells in 35-mm dishes at ~80% confluency (~8 × 10^5^ cells/dish) which were then infected with BCoV at a multiplicity of infection of 5 PFU per cell. For replication assay, supernatant fluids were harvested at 48 hpt and 500 µl was used to infect freshly confluent HRT-18 cells in a 35-mm dish (VP1). Total cellular RNA was extracted with TRIzol (Invitrogen) at 48 hpi of VP1 and 10 µg of extracted RNA was used for Northern assay. For internal controls, 3 µg of DI RNA transcript was transfected into BCoV-infected HRT cells and total cellular RNA was extracted with TRIzol at 4, 8, and 21 hpt of virus passage 0 (VP0) as indicated in Figure 6C and 10 µg of extracted RNA was used for electrophoresis in a formaldehyde-agarose gel. RNA was transferred from the gel to Nytran membrane by vacuum blotting and blots were probed with 5’-end ^32^P-labeled oligonucleotides. For detecting DI RNA specifically, the reporter-detecting oligonucleotide probe named TGEV(+), 5’ CATGGCACCATCCTTGGCAACCCAGA3’, was used. For detecting viral N subgenomic mRNA, viral M subgenomic mRNA, and the viral RNA portion of DI RNA the oligonucleotide named BCoVN(+), 5’ CCAGAACGATTTCCAAAGGACGCTCT3’, was used. For detecting the His-tag-encoding sequence, the oligonucleotide His(+), 5’ GTGGTGGTGGTGGTGGTG3’, was used. For detecting 18S rRNA, the oligonucleotide 18S rRNA(+), 5’ GCCTGCTGCCTTCCTTGGATCTGGTAGCC3’, was used. The probed blot was exposed to Kodak XAR-5 film at -80°C. For quantitating DI RNA, N subgenomic mRNA and 18S rRNA, probed blots were read with a Packard InstantImager Autoradiography System.
